# Potential Benefits of Nrf2/Keap1 Targeting in Pancreatic Islet Cell Transplantation

**DOI:** 10.3390/antiox9040321

**Published:** 2020-04-16

**Authors:** Alberto Jarrin Lopez, Hien Lau, Shiri Li, Hirohito Ichii

**Affiliations:** Department of Surgery, University of California, Irvine, CA 92868, USA; ajarrinl@uci.edu (A.J.L.); hlau2@uci.edu (H.L.); shiril@uci.edu (S.L.)

**Keywords:** Nrf2/Keap1, antioxidant, diabetes, pancreatic islet, transplantation

## Abstract

Permanent pancreatic islet cell destruction occurs in type 1 diabetes mellitus (T1DM) through the infiltration of inflammatory cells and cytokines. Loss of β-cell integrity secondary to oxidation leads to an inability to appropriately synthesize and secrete insulin. Allogenic islet cell transplantation (ICT) has risen as a therapeutic option to mitigate problematic hypoglycemia. Nevertheless, during the process of transplantation, islet cells are exposed to oxidatively caustic conditions that severely decrease the islet cell yield. Islet cells are at a baseline disadvantage to sustain themselves during times of metabolic stress as they lack a robust anti-oxidant defense system, glycogen stores, and vascularity. The Nrf2/Keap1 system is a master regulator of antioxidant genes that has garnered attention as pharmacologic activators have shown a protective response and a low side effect profile. Herein, we present the most recently studied Nrf2/Keap1 activators in pancreas for application in ICT: Dh404, dimethyl fumarate (DMF), and epigallocatechin gallate (EGCG). Furthermore, we discuss that Nrf2/Keap1 is a potential target to ameliorate oxidative stress at every step of the Edmonton Protocol.

## 1. Introduction

Type 1 diabetes mellitus (T1DM) leads to permanent pancreatic islet cell destruction via the infiltration of inflammatory cells and cytokines [1,2]. T1DM typically occurs in children and adolescents that have a genetic predisposition and have experienced an environmental stressor (i.e., virus or toxin) [3,4]. Under physiological conditions, β-cells elegantly uptake and convert glucose into ATP, stimulating ion gradients that drive the depolarization-dependent release of insulin. Loss of β-cell integrity leads to an inability to appropriately synthesize and secrete the peptide hormone. This is caustic metabolically, as the tissues are unable to meet their energy demands. In the United States, an estimated 30.3 million people of all ages had diabetes in 2015 [5], with approximately 5% of these having T1DM. Within those 30.3 million, it was estimated that 17,900 were children or adolescents younger than 20 years of age [5]. While the majority of diabetes cases are type 2 diabetes mellitus (T2DM), late-term T2DM is functionally equivalent to T1DM [6,7]. Allogenic islet cell transplantation (ICT) has thus risen as a therapeutic option to mitigate problematic hypoglycemia [8]. Following the Edmonton protocol, islet cells are procured from cadaveric donors and perfused into the hepatic portal vein [9].

Islet cells are at a baseline disadvantage as they lack a robust anti-oxidant defense system. In fact, relative to other tissues in the rat model, islet cells contain significantly less glutathione peroxidase, superoxide dismutase, and catalase [10,11]. The cells’ ability to combat oxidative stress is the key for remaining functional as reactive oxygen species (ROS) directly impact the integrity of the β-cell at the biomolecular level [12]. Higher sensitivity to stress along with pancreatic islet during isolation, preparation, and reperfusion-related injuries set the islet cells for engraftment and survival failure. It is no surprise then that the five-year insulin independence rate for allogenic ICTs is 25–50% [13,14,15,16]. Studies have further confirmed that nearly 15–50% of the approximated 1–1.5 million islet cells are lost in the isolation process [17], with transplants often times requiring three to four pancreases to achieve euglycemia [18]. Research efforts have accordingly begun focusing on ways to increase the antioxidant response. Particular attention has been given to the Nrf2/Keap1 pathway, a master regulator of antioxidant genes [19].

## 2. Nrf2/Keap1 Signaling Pathway

**Figure 1:**
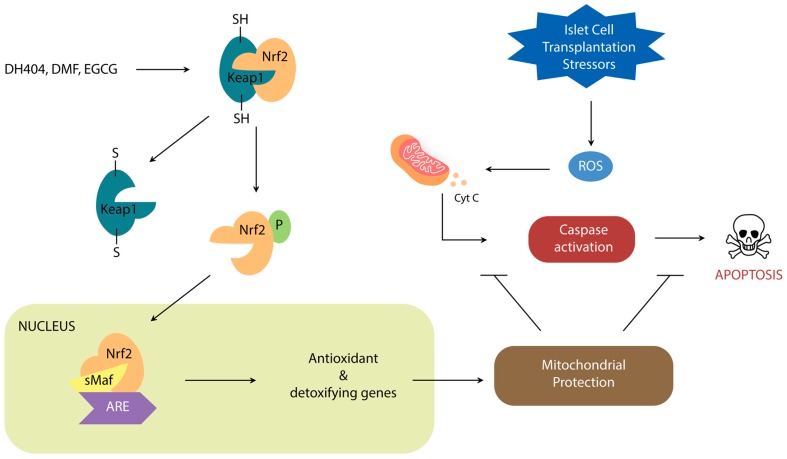
Schematic demonstrating the role of Nrf2/Keap1 signal transduction on oxidative stress regulation.

Nrf2 is a leucine zipper protein that is covalently repressed by its regulator Keap1 in the cytoplasm through ubiquitination [20,21,22]. Stress induces the separation of these two molecules via modification of the Keap1 cysteine residues, allowing Nrf2 to enter the nucleus and heterodimerize with small Maf Proteins (MafP) (Figure 1). The Nrf2/MafP complex binds to genes which promote the transcription of multiple antioxidant enzymes [23,24,25,26,27]. Yagishita et al. have demonstrated that the Nrf2/Keap1 system plays a critical role in the protection of pancreatic β-cells from oxidative damage through repressed apoptosis and enhanced proliferation [28]. Nrf2/Keap1 thus promises a potential way of reducing the oxidative damage that occurs in ICT.

Dh404, dimethyl fumarate (DMF), and epigallocatechin gallate (EGCG) are the Nrf2 activators that have been most studied in context of pancreatic inflammation and islet cell transplantation.

### Nrf2 Activators

Dh404, formally CDDO-9,11-dihydro-trifluoroethyl amide (CDDO-dhTFEA) [29] is a synthetic oleanane triterpenoid (SO) plant derivative used in oriental medicine [30] for anti-inflammatory and anti-tumorigenic purposes [31,32]. Since Yates et al.’s first report of dh404 as a protective agent against aflatoxin-induced tumorigenesis [33], dh404 has been studied in oncology, chronic renal disease, and recently in diseases of the pancreas. As a group, SOs are unique because they are one of the most potent inducers of the Nrf2 pathway with selective induction of phase II detoxifying and antioxidant enzymes [34].

Dh404 has proven to be protective in the pathogenesis of acute pancreatitis. Dh404 (1 mg/kg)-treated rats 24 h before L-arginine (600 mg/100 g)-induced pancreatitis showed reductions in inflammatory cells, acinar structural damage, edema, necrosis (*p* < 0.001), and rates of apoptosis (*p* < 0.05) [35]. Malondialdehyde (MDA), which is an indicator of lipid peroxidation, was also reduced (*p* < 0.05) [35]. MDA was further shown to be lower in dh404-cultured cells compared to controls when in 200 μM H_2_O_2_ for a 24-h period [35]. Lastly, the effect of dh404 was shown to be temporally dependent, as cells that were incubated with 500 nM dh404 for 1 h had nearly twice the intranuclear Nrf2 concentration as cells incubated for 30 min. When dh404 treatment was prolonged for 24 h, the presence of anti-oxidant enzymes such as Heme Oxygenase-1 (HO-1), superoxide dismutase (SOD), and catalase (CAT) was recorded [35].

The mechanism of dh404-mediated Nrf2 activation is not yet clear. Ichikawa et al. showed that dh404 is involved in a unique interaction with Cys-151 of Keap1, which under physiological conditions binds Cul3/Rbx1 E3 ligase complex to target Nrf2 ubiquitination and subsequent digestion [36]. On another hand, Li et al. have shown that dh404-mediated Nrf2-activated pathway involves the autophagy of toxic ubiquitinated proteins driven directly by Nrf2 induction, and not by ROS [37]. Because ROS were previously shown [38] to endogenously drive the autophagy process as a defense mechanism to inflammation, these findings suggest that dh404 activates Nrf2 to simultaneously carry out two actions that are not mutually exclusive. Whether this response is entirely due to the Nrf2 or supplemented by an additional pathway activated by dh404 necessitates further investigation.

Dimethyl fumarate, otherwise known as BG-12 or Tecfidera, is a fumarate ester that started out as a recognized anti-carcinogen [39], in the 1990s it was licensed in Germany for treatment of psoriasis, and more recently in 2013 has received approval by the US Food and Drug Administration (FDA) for the treatment of relapsing-remitting multiple sclerosis [40]. Our lab examined the role of DMF as a Nrf2 activator in the setting of pancreatitis [41,42]. Pancreata of rats treated with DMF (25 mg/kg) 24 h prior to L-arginine (3 g/kg)-induced acute pancreatitis showed reductions in the severity of inflammatory cell infiltration, acinar damage, perilobar edema, and cell necrosis (*p* < 0.001) [41]. Similarly, rats that were orally fed DMF (25 mg/kg) prior to and after L-arginine-induced-chronic pancreatitis resulted in improved glucose tolerance, better-preserved tissue architecture (less atrophy, edema, and fatty infiltration) (*p* < 0.05), significantly lower levels of inflammatory markers (myeloperoxidase (MPO) and MDA), and significantly higher expression of antioxidants (i.e., HO-1) [42]. Zhang et al. corroborated similar findings and also demonstrated that animals transplanted with DMF-treated-cells had lower blood glucose (*p* < 0.01) and preserved β-cell function [43].

Interestingly, and conveniently, DMF has demonstrated to be most efficacious under stressful conditions. In a study performed by Schultheis et al., islet cells from adult mice were cultured for 12-16 h in DMF, and then for 2 or 48 h under control or glucolipotoxic conditions (25 mmol/L glucose and 100 µmol/L palmitate) [44]. Compared to controls, cells in the glucolipotoxic medium had a decrease in oxidized status, superior insulin secretion, and a higher mitochondrial membrane potential (50 vs. 10 µmol/L) at 48 h [44]. While the benefits of DMF in the treatment of inflammatory conditions have been shown to be due to a sundry of anti-inflammatory responses [45,46,47,48], the specific mechanism behind Nrf2-activation necessitates further investigation.

Epigallocatechin gallate is a main ingredient of green tea and has been described since the 1990s to have anticarcinogenic, antioxidant, antiangiogenic, antiviral properties, and more recently antidiabetic properties [49,50,51,52]. It has been shown to act as a neutralizing agent for ROS, and to have anti-inflammatory effects that have reduced liver fibrosis [53] and even contribute to hepatic regeneration [54].

EGCG has been shown to suppress cytokine-induced pancreatic β-cell damage in vitro. Pretreatment of RINm5f cells with EGCG (0–200 µg/mL) in presence of proinflammatory cytokines resulted in no cell apoptosis compared to the 55% that became apoptotic in the absence of EGCG [55]. In fact, the response was noted to be concentration-dependent, with 200 µg/mL EGCG nearly fully blocking the cell death response, abrogating the three-fold increase in NO_2_ seen in control, and completely inhibiting the production of inducible NO synthase (iNOS) [55]. To simulate the inflammatory environment of T1DM in vivo, the authors induced autoimmune diabetes with a 250 mg/kg streptozotocin (STZ) dose in C57BL/KsJ mice for five consecutive days. In the experimental group, EGCG (100 mg/kg) was administered daily with STZ, and alone for the five days thereafter [56]. Relative to the control group, the EGCG-treated mice had a significantly reduced STZ-induced hyperglycemia, and markedly suppressed iNOS mRNA expression [56].

Despite current evidence that EGCG is an activator of the Nrf2/Keap1 pathway [57], the exact mechanism of how this occurs has yet to be elucidated. There is evidence that the activation involves the mitogen-activated protein kinase (MAPK) cascade [58], electrophilic interactions with the cysteine residues in Keap1 [59], as well as ROS-derived auto-oxidation of EGCG facilitating the release of Nrf2 from its complex [60].

Having introduced the activators with most promise in the setting of pancreatic islet cell transplantation, next we discuss how the Nrf2/Keap1 pathway is a potential target at each step of the transplantation process to protect islet cells from oxidative damage.

## 3. Nrf2 Roles in Steps of Islet Transplantation

### 3.1. Nrf2/Keap1: A Target for Pre-Transplant Protection of Islet Cells

#### 3.1.1. Isolation: Metabolic Challenges

Ischemia poses a particular challenge to islet cells because of the cells’ low antioxidant defenses and lack of glycogen reserves which prevents them from producing ATP (critical for insulin secretion) [61].

The Nrf2 activator dh404 has shown protective benefits when used during the process of islet cell isolation [62]. Rats that were fed dh404 (0.6 mg/kg body weight) once daily for three days prior to islet cell isolation had greater islet cell yield (*p* < 0.05) and β-cell content (*p* < 0.05) compared to vehicle-treated rats [62]. HO-1 upregulation was 10-fold higher and the percentage of apoptotic cells was lower (*p* < 0.05) in treated cells. The same authors have also highlighted that the capacity of the Nrf2 response is dependent on the stress state of the cell [63]. When human islets were treated with 500 nM dh404, a significantly higher proportion of β-cell survival was observed in the presence of 200 µM H_2_O_2_ when compared to those not treated with dh404 (74% vs. 57%; *p* < 0.05) [63]. Within the cells that survived, they also noted higher levels of nuclear Nrf2 and higher mRNA levels of antioxidant genes like NAD(P)H dehydrogenase [quinone] 1(NQO1), HO-1, and Glutamate-cysteine ligase catalytic subunit (GCLC) [63].

Tetrahydrocurcumin (THC), the final hydrogenated metabolite of curcumin (active ingredient of *Curcuma longa* L.), has demonstrated very strong antioxidant properties. In an effort to decrease the ROS-mediated islet cell damage prior to transplantation, Kim et al. cultured Balb/c mice islet cells for 24 h in a THC-supplemented medium, and exposed them to various cytokines [64]. They found that THC-treated cells had 1.3-fold greater glucose sensitivity and produced B-cell lymphoma 2 (BCL2) (antiapoptotic) along with protective caspase proteins [64]. Although at the time of the study it was established that THC increased glutathione (GSH) (a low molecular weight antioxidant), Luo et al. have recently demonstrated that this occurs specifically through the Nrf2/Keap1 pathway [65]. THC causes Nrf2 to translocate into the nucleus, and induce glutamate cysteine ligase (GCL), which is a key determinant of GSH synthesis [66]. Whether THC is a broad antioxidant activator (like dh404) or a more selective one remains to be answered.

#### 3.1.2. Preservation: Dynamic Temperature Stress on Islet Cells

**Figure 2:**
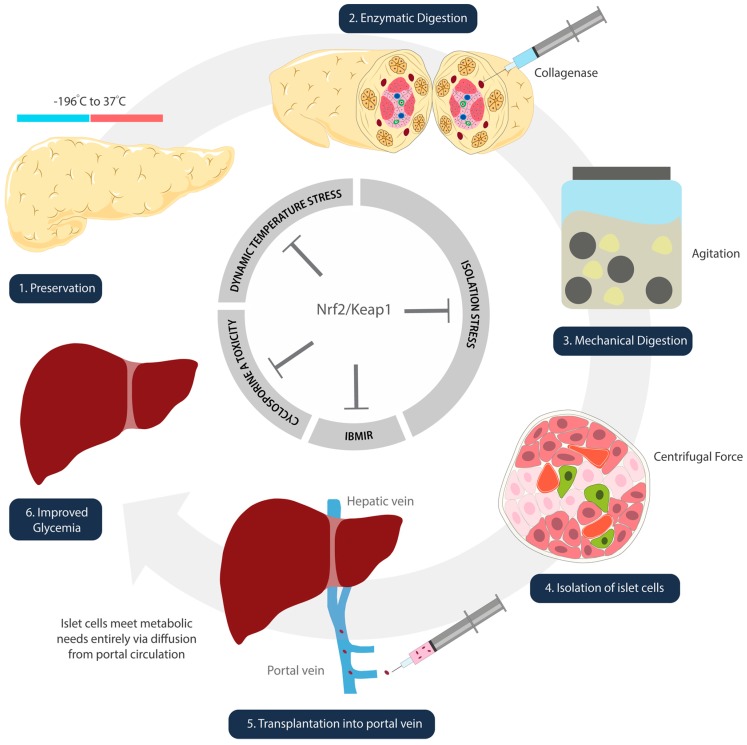
Schematic illustration denoting steps of islet cell transplantation. Nrf2/Keap1 pathway activation at each of these steps has the potential to ameliorate oxidative damage that occurs as a result of temperature (1), isolation (2–4), reperfusion (5), and immunosuppression side-effect-mediated insults.

Islet cells experience oxidative stress in the freezing and thawing processes that take place during isolation and preparation (Figure 2) [67]. Kanitkar et al. have demonstrated the protective effect of curcumin against extreme temperatures [68]. Isolated mice islet cells were cryopreserved using a standard protocol. These cells were stored in liquid nitrogen for seven days, and thawed rapidly from –196 °C to 37 °C [68]. Experimental groups included islets treated with 10 µM curcumin during cryopreservation, during the 24 h post-thaw incubation period, and during both [68]. Inclusion of curcumin at both steps demonstrated increased yield, better morphology and integrity, enhanced basal and stimulated insulin secretion, as well as induction of Hsp70 and HO-1 [68]. While the study did not directly discuss the activation of Nrf2/Keap1, we infer its role given that curcumin is metabolized into THC, a confirmed activator [65].

The antioxidant response during cold-storage appears to be more protective than oxygenation alone by means of perfluorohexyloctane (F6H8) (a low specific density, oxygen carrier) [69]. Brandhorst et al. may have indirectly shown the involvement of Nrf2/Keap1 in their study where 5 mmol/L L-glutamine was administered to pig islet cells exposed to warm (30 min) and cold (3 h) storage [70]. Their hypothesis was based upon previous evidence that glutamine infusion led to formation of intra-islet GSH [71,72]. This was anticipated, as glutamate (deaminated glutamine) is a building block of GSH [73]. While the authors noted a protective effect, they could not explain why glutamine leads to GSH formation specifically at a time of stress. Recently, Sayin et al. have shown that tumor cells hyperactivate the Nrf2 system in order to maintain oxidative homeostasis [74]. In fact, they discovered that tumor cells depend heavily on exogenous glutamine for GSH synthesis [74]. In Brandhorst et al.’s study, because glutamine ameliorated the temperature-induced oxidative damage, it is possible that stress upregulated the Nrf2/Keap1 system, which, in effect, increased the demand for the supplied glutamine to synthesize GSH.

#### 3.1.3. Digestion and Isolation: Chemical and Mechanical Stress on Islet Cells

Pancreata digestion is required to extract the islet cells from the donor organ. It is a harsh process that involves chemical and mechanical exposures. The islet cells undergo collagenase digestion, agitation in marble glass, and centrifugal forces all while experiencing ischemia and temperature changes (Figure 2) [75]. Although there are no published studies on the direct effect of Nrf2 during pancreatic digestion, one study by Ito et al. indicates the likelihood of its involvement [76]. Pancreata removed from beagle dogs were treated with p38MAPK inhibitor (P38IH) prior to preservation, and were assessed after isolation [76]. The intraductal infusion of P38IH was found to reduce TNF-alpha expression, reduce B-cell apoptosis, and significantly improve the islet cell yield (76). Naidu et al.’s publication one year later elucidated that inhibition of p38MAPK upregulates HO-1 expression via activation of the Nrf2 pathway [77].

### 3.2. Nrf2/Keap1: A Target for Post-Transplant Protection of Islet Grafts

After isolation, the islet cells are injected into the portal vein and distribute heterogeneously in the liver’s peripheral branches [78]. It takes approximately 10 days [79] before islet cells establish revascularization, hence the liver is a desirable site due to the constant blood flow which the diffusion-limited islets heavily depend on. While hypoxia is certainly a limiting factor in the survival capacity of the cells once infused, Muthyala et al. showed that even when groups of islet cells were spaced in alginate microcapsules to effectively increase the surface area available for diffusion, neither metabolic activity nor insulin secretion differed significantly from those that were infused as free cells [80]. On the contrary, several groups have shown in both mouse and human models that tissue factor (TF) (expressed on the islet cell membrane) is a major player in the activation and release of a downstream inflammatory cascade that has been coined the immediate blood mediated inflammatory response (IBMIR). IBMIR consists of leukocyte infiltration, complement activation, and thrombosis which has been seen on MRI and positron-emission tomography monitoring to lead to a dramatic reduction of islet cells in the very early peri-transplant period [81,82,83,84]. Utilization of Kosinova et al.’s model for in vivo monitoring of liver ischemia in relation to pancreatic islet cell transplants shows promise as a system capable of quantifying the alleviating extent of inflammation [85] with Nrf2/Keap1 activation.

Wada et al. have for the first time shown that EGCG treatment of mouse islets in vivo improves viability and function through the activation the Nrf2 system [86]. To test the response of EGCG in context of cell transplantation, the 100 µM EGCG group (concentration found to promote significantly higher cell viability) and a control group were transplanted under the kidney capsule of a 200 mg/kg STZ-induced diabetic mouse [86]. The cytoprotective effect of EGCG was evident as the ROS production was lower (*p* < 0.01), and the HO-1 mRNA expression of the nuclear-translocated Nrf2-positive cells was higher (*p* < 0.05) in treated cells [86]. Of note, while 100 µM was established as the optimal concentration for cell viability and stimulation index, higher concentrations actually proved harmful [86]. The viability and function tests of islets treated with 500 µM EGCG were significantly lower than those treated with 100 µM [86].

While a majority of the studies on Nrf2 activators have revolved around their protective role in acute and chronic pancreatitis, there are currently no published studies demonstrating their antioxidant effect in context of the immediate reperfusion period after islet cell transplantation. Nevertheless, liver and renal tissue damage secondary to ischemia/reperfusion has been studied in the presence of Nrf2 activator pretreatment, and the results are promising. Our group looked at the effect of DMF pretreatment in rats that were subjected to ischemia for 1 h and reperfusion for 2 h [87]. Rats that received orally-administered DMF (25 mg/kg, 2x/day) pretreatment had a significant decrease in levels of MDA (*p* = 0.0009), increased expression of CAT (*p* = 0.03) and Glutamate-Cysteine Ligase Modifier Subunit (GCLM) (*p* = 0.04), decreased neutrophils and markers of inflammation, superior endothelial function and histopathological integrity (*p* = 0.02), and increased ATP levels (apoptosis consumes NAD^+^) (*p* = 0.02) [87].

Yoon et. al. looked at the effect of sulforaphane pretreatment in Hexokinase 2 (HK2) renal tubular epithelial cells incubated in anaerobic jars at 37 °C, and observed a concentration-dependent benefit [88]. Sulforaphane pretreatment at 20 µM improved survival to nearly 93% (vs. 57% in control), and increased HO-1, NQO1, Glucocorticoid receptor (GR), and glutathione peroxidase (GPx) mRNA [88]. In vivo pretreatment resulted in significantly depressed (*p* < 0.01) serum creatinine and less macroscopic histological evidence of tubular damage by a factor of 10 relative to untreated controls [88].

The potential application of Nrf2 activators extends beyond the peri-transplant period given evidence of Nrf2′s ability to prevent the side-effects caused by immunosuppressants [89]. Our group has previously published that DMF treatment confers renal protection against cyclosporine A nephrotoxicity [89]. Given that transplant patients require long-term immunosuppression with calcineurin inhibitors, we postulate that Nrf2/Keap1 pathway activation plays a critical role in the long-term wellbeing of our patients as well. Future studies are needed to determine whether certain activators will act and be tolerated better as adjuncts to immunosuppressive therapy than others.

## 4. Discussion

Activation of the Nrf2/Keap1 pathway has been demonstrated to confer antioxidant protection in every step of the islet cell transplantation. This is not surprising as the cell is exposed to taxing conditions every step of the way. While dh404, DMF, and EGCG have all been described to have good safety profiles, it is important to note that such is the case when the appropriate dose is used. Many studies have proven the dose-dependent effects of Nrf2 activators. Thus far, dh404 has proven to have an optimal concentration in vivo at 500 nM, DMF at 0.25–5 µM, and EGCG at 100 µM. When the concentrations are too low or too high relative to their optimum, oxidative stress eventually leads to cell death [86,90,91]. Thus, further confirmatory testing at optimal dose of those medications in different applications are necessary prior to human experimentation. Furthermore, the context of application for these Nrf2 activators must be determined as it has been described that they are most effective under stress. Whether the recipient may benefit from Nrf2 activators prophylactically to achieve antioxidant protection prior to transplant would also be worth investigation.

While dh404, DMF, and EGCG are the Nrf2 activators that have been most studied in context of pancreatic inflammation and islet cell transplantation, it is important to note that there are more Nrf2 activators that have been described [92,93]. Establishing a profile index of which activator is most effective and safe would be prudent. Since different activators appear to have unique mechanisms of Nrf2 activation, the simultaneous use of multiple types of activators might result in a summative response. Once the appropriate Nrf2 activator(s) and their optimal dose(s) are confirmed, the next step will be to utilize such activators during recognized oxidative-heavy steps of the peri-transplant process.

## 5. Conclusions

The antioxidant response promulgated by the activation of the Nrf2/Keap1 pathway offers a potential protective mechanism during pancreatic islet cell transplantation. By reducing the amount of oxidative damage islet cells experience in the preservation, isolation, and transplantation process, Nrf2 activators promise a means to enhance viability and henceforth prolong the longevity of transplanted islet cells in Type 1 diabetic recipients.
